# Applicability and performance of EUCAST’s rapid antimicrobial susceptibility testing (RAST) on primarily sterile body fluids in blood culture bottles in laboratory routine with total lab automation

**DOI:** 10.1007/s10096-020-04146-6

**Published:** 2021-01-12

**Authors:** Jasmin Kaur Jasuja, Stefan Zimmermann, Irene Burckhardt

**Affiliations:** Department for Infectious Diseases, University Hospital Heidelberg, Im Neuenheimer Feld 324, 69120 Heidelberg, Germany

**Keywords:** RAST, EUCAST, Primarily sterile body fluids, Blood cultures, TLA

## Abstract

Optimisation of microbiological diagnostics in primarily sterile body fluids is required. Our objective was to apply EUCAST’s RAST on primarily sterile body fluids in blood culture bottles with total lab automation (TLA) and to compare results to our reference method Vitek2 in order to report susceptibility results earlier. Positive blood culture bottles (BACTEC™ Aerobic/Anaerobic/PEDS) inoculated with primarily sterile body fluids were semi-automatically subcultured onto Columbia 5% SB agar, chocolate agar, MacConkey agar, Schaedler/KV agar and Mueller-Hinton agar. On latter, cefoxitin, ampicillin, vancomycin, piperacillin/tazobactam, meropenem and ciprofloxacin were added. After 6 h, subcultures and RAST were imaged and MALDI-TOF MS was performed. Zone sizes were digitally measured and interpreted following RAST breakpoints for blood cultures. MIC values were determined using Vitek2 panels. During a 1-year period, 197 *Staphylococcus aureus*, 91 *Enterococcus* spp., 38 *Escherichia coli*, 11 *Klebsiella pneumoniae* and 8 *Pseudomonas aeruginosa* were found. Categorical agreement between RAST and MIC was 96.5%. Comparison showed no very major errors, 2/7 (28.6%) and 1/7 (14.3%) of major errors for *P. aeruginosa* and meropenem and ciprofloxacin, 1/9 (11.1%) for *K. pneumoniae* and ciprofloxacin, 4/69 (7.0%) and 3/43 (5.8%) for *Enterococcus* spp. and vancomycin and ampicillin, respectively. Minor errors for *P. aeruginosa* and meropenem (1/8; 12.8%) and for *E. coli* and ciprofloxacin (2/29; 6.5%) were found. 30/550 RAST measurements were within area of technical uncertainty. RAST is applicable and performs well for primarily sterile body fluids in blood culture bottles, partially better than blood-based RAST. Official EUCAST evaluation is needed.

**Supplementary Information:**

The online version contains supplementary material available at 10.1007/s10096-020-04146-6.

## Introduction

The improved diagnosis of causative pathogens in primarily sterile body fluids is an important but difficult goal to achieve in the microbiological field. Gram staining from native specimens is often non-contributory and even standard cultivation can miss microbial organisms despite specific clinical signs [1, 2]. For this, the addition of primarily sterile body fluids, e.g. joint, pleural or peritoneal fluids, to blood culture media has significantly improved and accelerated the yield of causative pathogens [2, 3]. Furthermore, poly-microbial infections particularly with *methicillin-resistant Staphylococcus aureus* (MRSA), *P. aeruginosa* and *Enterococcus* spp. are diagnosed with higher sensitivity [3]. Indeed, such pathogens are of special clinical relevance regarding appropriate antimicrobial therapy. As Zelenitsky et al. showed most common and significant organisms causing peritoneal-dialysis-related peritonitis like *S. aureus*, *E. coli* and *K. pneumoniae* have increased resistance patterns against commonly used antibiotics such as methicillin and ciprofloxacin compared to former elicitation [4]. Even Kitterer et al. demonstrated rising resistance leading to a change in the choice of first line therapy [5]. This is why rapid ID and rapid antimicrobial susceptibility testing (RAST) is of significant interest, even for primarily sterile body fluids. Tian et al. performed rapid microbial identification via MALDI-TOF MS and rapid multiple AST i.a. directly from positive primarily sterile body fluids inoculated in blood culture medium, but correct ID for Gram-positive bacteria was only achieved in 87.2% [6]. Though rapid multiple AST via Vitek AST system was successful, the average time to report was ≥ 8 h, which is incongruent to the definition of rapid AST [6, 7].

We already successfully implemented EUCAST’s RAST on positive blood culture bottles with total lab automation (TLA, BD Kiestra™) in our laboratory routine [8]. In the current study, we investigated the applicability and performance of EUCAST’s RAST on primarily sterile body fluids inoculated in blood culture bottles with TLA in clinical practice. Our aim was to explore if EUCAST’s RAST is applicable on primarily sterile body fluids by comparing non-blood-based RAST results with our routine reference Vitek2 to check if categorical results can be reported earlier by RAST and appropriate antibiotics can be applied in time.

## Material and methods

### Settings

The study was performed between 1st November 2018 and 30th November 2019 at the Department for Infectious Diseases at the University Hospital Heidelberg, Germany. Our analysis included BD BACTEC™ Plus Aerobic/F, BD BACTEC™ Plus Anaerobic/F and BD BACTEC™ PEDS Plus/F blood culture bottles inoculated with primarily sterile body fluids sent during the aforementioned study period. Blood culture bottles inoculated with blood were excluded. Each bottle was analysed individually. The following methods were introduced during the study period and since then performed routinely. After arrival at our laboratory, aerobic or PEDS blood culture bottles were inoculated with 2 ml of BD BACTEC™ FOS Kit and incubated in the BD BACTEC™ FX instrument for up to 5 days or until they signalled positive [9]. Joint fluid was regularly incubated for 14 days or until flagged as positive.

Each positive bottle was processed in the semi-automatic part of our TLA by simultaneous Gram staining, subculturing and preparing RAST. Gram slides were prepared and stained manually and examined under microscope by a physician. Microscope results were sent as preliminary electronic report to the ward.

**Fig. 1:**
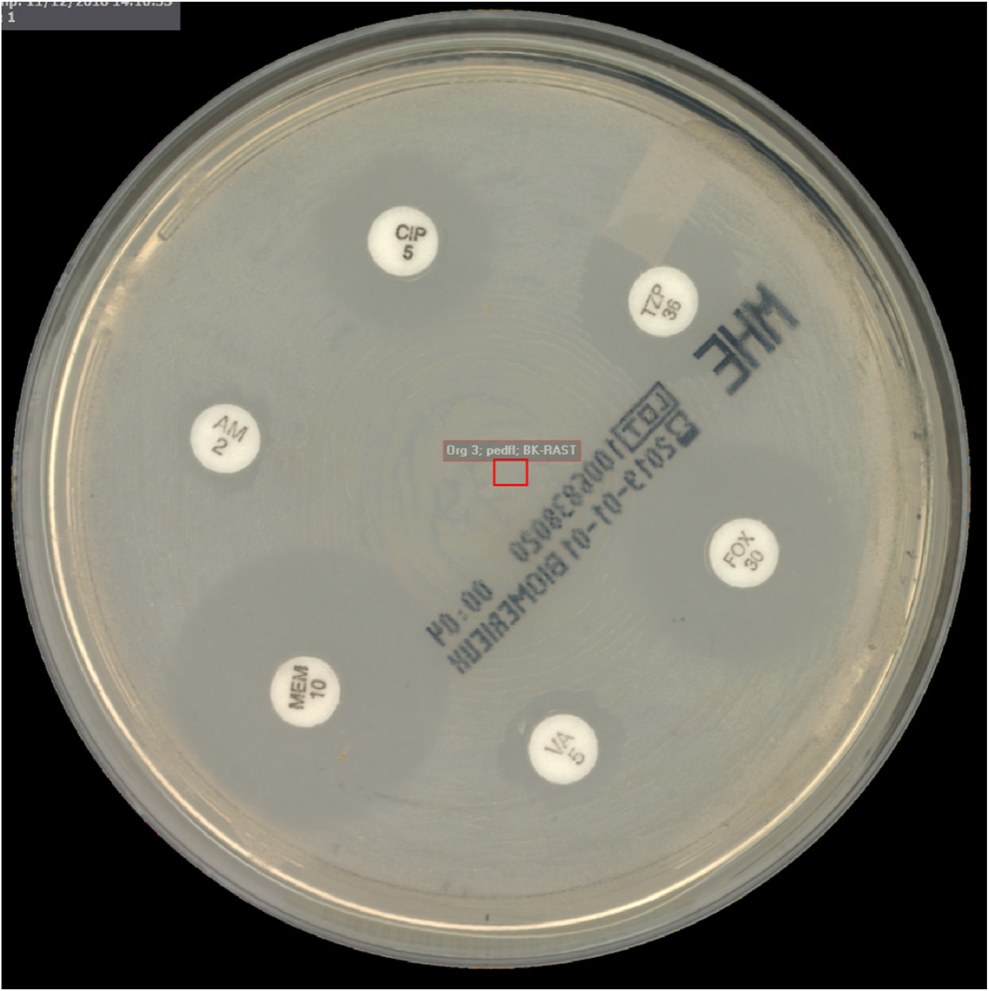
RAST image of *Staphylococcus aureus* isolated from joint fluid after 6 h on Mueller-Hinton agar with visible zone diameters taken by a total lab automation (TLA) at the Department for Infectious Diseases at the University Hospital Heidelberg, Germany. As soon as a blood culture bottle inoculated with primarily sterile body fluid flagged as positive i.a. rapid antimicrobial susceptibility testing (RAST) was prepared on a Mueller-Hinton agar as established by EUCAST for blood-based RAST. After 6 h, automatic imaging was done by TLA. Images were digitally viewed by a technician and zone diameters were measured (not measured here) (CIP, ciprofloxacin; TZP, piperacillin/tazobactam; FOX, cefoxitin; VA, vancomycin; MEM, meropenem; AM, ampicillin)

Subcultures on blood agar (Columbia agar, 5% sheep blood, BD), chocolate agar (bioMérieux), MacConkey agar (bioMérieux) and in case of an anaerobic bottle additionally on Schaedler/KV agar (5% sheep blood, BD) were done. RAST was prepared following EUCAST’s methodology for positive blood cultures bottles. Therefore, 150 μl of primarily sterile body fluid in blood culture bottle was subcultured on a Mueller-Hinton agar (bioMérieux) and six discs of commonly used antimicrobials, namely cefoxitin (30 μg, BD), ampicillin (2 μg, BD), vancomycin (5 μg, BD), piperacillin/tazobactam (30/6 μg, BD), meropenem (10 μg, BD) and ciprofloxacin (5 μg, BD), were applied (Fig. 1). Streaking via magnetic rolling bead technology was done by TLA. Afterwards, aerobic plates including RAST subculture were transferred to the incubators (35 °C, O_2_: RAST plate, 5% CO_2_: blood agar, chocolate agar, MacConkey agar) of the TLA (ReadA Compact), while the anaerobic plate was incubated in an anaerobic jar in an external incubator. All plates were automatically imaged by TLA after 6 h and 23 h (latter except RAST). Anaerobic plates were viewed manually. MALDI-TOF MS (Microflex and Smart, Bruker Daltonik GmbH, Bremen), RAST reading and preparation of Vitek2 were done with 6 h growth. Inhibition zones were digitally viewed, measured by positioning zone circles using TLA software and interpreted following the EUCAST RAST guidelines (version 1.0 and 1.1). An electronic report with ID and preliminary AST with RAST results was sent to the ward. On the next day, an electronic report with ID and final MIC results obtained from Vitek2 was sent.

In case of blood culture bottle signalling positivity in the late afternoon, images of subcultures and RAST were taken outside operational time (7 am–6 pm on weekdays and 7 am–4 pm on weekends) and were interpreted in the next morning after performing MALDI-TOF MS. RAST was electronically reported afterwards and Vitek2 was prepared for the upcoming day.

The terms of categorical agreement, very major errors (VME), major errors (ME) and minor errors (MinE) as recommended by Cumitech were applied [10]. Originally, an error is declared as very major error (VME) when the new AST is susceptible but the reference method results in resistant response. Major errors (ME) are declared with a resistant response in the new AST while the reference method indicates a susceptible response. Minor errors (MinE) are observed when either the new AST or the reference method indicates an intermediate response and the other one a susceptible or resistant response, respectively. As we could not perform microdilution in our daily routine, we took Vitek2 as reference method. Hence, differences can also be referred as discrepancies but we continue with the generally accepted term ‘error’ and the recommended categories.

Since there is no intermediate category for RAST, EUCAST introduced the concept of ‘area of technical uncertainty’ (ATU) where interpretation to susceptible or resistant result is not possible. Hence, ATU results were not included for MinE calculation and could only arise when comparing susceptible and resistant RAST to intermediate Vitek results. Accepted percentage for categorical agreement was ≥ 90%. VME and ME rate was supposed to be ≤ 3%, respectively. A combined performance rate of ≤ 7% for ME and MinE rate was recommended. VME, ME and MinE rates were calculated for each drug and drug-species combination, respectively. For data analysis, we compared RAST with our reference method Vitek2 to check if RAST can predict final MIC results so that clinicians may adapt antimicrobial therapy earlier.

### Statistical analysis

Data on RAST and MIC results were obtained from our LIS (SwissLab, Nexus AG) and analysed with Microsoft Excel 2010.

## Results

During the study period from 1st November 2018 to 30th November 2019, a total of 5341 blood culture bottles inoculated with primarily sterile body fluids were processed in our laboratory routine. Thereof, 937 (17.5%) bottles signalled positive. A total of 64 bottles (6.8%) were excluded due to poly-microbial growth which were detected with the 6 h growth and RAST was not reported. A total of 13 bottles (≤ 1%) were sorted out due to false-positive signal. Hence, 345 (of 860) positive mono-bacterial blood culture bottles filled with primarily sterile body fluids with readable zone diameters and available RAST breakpoints were eligible. Most of the primarily sterile body fluids contained joint fluid (*n* = 223), ascites (*n* = 52) and dialysate (*n* = 22). A total of 515 bottles contained pathogens, which do not yet have EUCAST RAST breakpoint criteria; for further analysis, see Table 1.

**Table 1:** Overview of all sent blood culture bottles inoculated with primarily sterile body fluids, positive signalled bottles and RAST-eligible pathogens within the time period of 1st November 2018 to 30th November 2019 at the Department for Infectious Diseases at the University Hospital Heidelberg, Germany. A total of 5341 aerobic, anaerobic and PEDS blood culture bottles inoculated with various primarily sterile body fluids were sent of which 937 bottles flagged as positive. A total of 345 positive blood culture bottles were eligible for RAST and comparison to MIC results. In total, 550 drug-species measurements were analysed

	*n*
Overall sent blood cultures inoculated with primarily sterile body fluids	5341
Flagged as positive	937
- False-positive bottles	13
- Blood culture bottles with ≥ 2 pathogens	64
Positive blood culture bottles with mono-bacterial growth	860
Pathogens for which RAST was applicable	345
- BACTEC™ Aerobic	108
- BACTEC™ Anaerobic	150
- BACTEC™ PEDS	87
Thereof analysed drug-species measurements	550
Type of primarily sterile body fluids	
- Joint fluid	223
- Ascites	52
- Dialysate	22
- Pleural fluid	14
- Easy flow drainage	12
- Other various drainage fluids (thoracic drainage, pericardium drainage, liver abscess, not-inscribed drainage)	20
- Gall bladder puncture	2
*S. aureus*	197
- *MSSA*	183
- *MRSA*	14
*Enterococcus* spp.	91
- *E. faecalis*	41
- *E. faecium*	28
- *Vancomycin-resistant E. faecium* (*VRE*)	22
*E. coli*	38
*K. pneumoniae*	11
- Carbapenemase-producing *K. pneumoniae*	1
*P. aeruginosa*	8
Other species than those validated for RAST	572
*S. epidermidis*	211
Other coagulase-negative *Staphylococcus* spp*.* (*S. capitis*, *S. caprae*, *S. cohnii*, *S. haemolyticus*, *S. hominis*, *S. lugdunensis*, *S. petrasii*, *S. pettenkoferi*, *S. saccharolyticus*, *S. warneri*, *S. simulans*)	135
*Streptococcus* spp. (*S. agalactiae*, *S. anginosus*, *S. canis*, *S. constellatus*, *S. dysgalactiae*, *S. gallolyticus*, *S. gordonii*, *S. intermedius*, *S. lutetiensis*, *S. mitis*, *S. oralis*, *S. parasanguinis*, *S. pyogenes*, *S. salivarius*, *S. sanguinis*, *S. thermophiles*, *S. vestibularis*, *S. pneumoniae*, *S. viridans*)	85
*Other Enterococcus* spp. (*E. gallinarum*, *E. avium*)	13
*Other gram-positive cocci* (*Aerococcus viridans*, *Micrococcus luteus*, *Parvimonas micra*, *Peptoniphilus harei*, *Rothia* (*Stomatococcus*) *mucilaginosa*, *Ruminococcus gnavus*)	8
Gram-positive bacilli (*Actinomyces funkei*, *Actinomyces neuii*, *Arthrobacter sanguinis*, *Bacillus cereus complex*, *Bacillus* spp., *Brevibacterium paucivorans*, *Clostridium* spp., *Corynebacterium* spp., *Cutibacterium* spp., *Gordonia polyisoprenivorans*, *Lactobacillus rhamnosus*, *Nocardia farcinica*, *Paenibacillus phoenicis*)	54
Other *Enterobacterales* (*Citrobacter* spp., *Enterobacter cloacae complex*, *Hafnia alvei*, *Klebsiella* spp., *Morganella morganii*, *Proteus* spp., *Providencia stuartii*, *Serratia* spp.)	7
Other gram-negative pathogens (*Acinetobacter* spp., *Aeromonas caviae*, *Bacteroides fragilis*, *Burkholderia cepacia complex*, *Haemophilus influenzae*, *Moraxella* spp., *Pantoea* spp., *Pseudomonas alcaligenes*)	17
*Candida* spp.	42

For 345 bottles the categorical interpretations (susceptible/resistant) of RAST were compared to the respective Vitek2 results (susceptible/susceptible, increased exposure/resistant) (Table 2). That included 197 *Staphylococcus aureus*, *91 Enterococcus spp*., 38 *Escherichia coli*, *11 Klebsiella pneumoniae* and 8 *Pseudomonas aeruginosa* and resulted in 550 individual drug-species measurements (197x cefoxitin and *S. aureus*, 91x ampicillin and 91x vancomycin and *Enterococcus* spp., 57x piperacillin/tazobactam, ciprofloxacin and meropenem and *E. coli*, *K. pneumoniae* and *P. aeruginosa* altogether). As recommended by EUCAST, ATU was not interpreted [11].

**Table 2:** A total of 345 positive blood culture bottles inoculated with primarily sterile body fluids were tested with EUCAST’s antimicrobial susceptibility testing (RAST) directly feasible from positive blood culture at the Department for Infectious Diseases at the University Hospital Heidelberg, Germany. Inhibition zones from RAST were compared to MIC results obtained from Vitek2. Very major errors (VME), major errors (ME) and minor errors (MinE) were calculated. The EUCAST RAST guidelines only contain an ‘area of technical uncertainty’ (ATU) instead of intermediate results. Zone diameters falling into the ATU category cannot be used to predict susceptibility or resistance. For determining VME and ME, isolates with ATU interpretation were excluded. MinE were only determined for susceptible and resistant RAST response to intermediate Vitek results (*S*, susceptible; *R*, resistant; *I*, susceptible, increased exposure; *ATU*, area of technical uncertainty)

		RAST interpretation			Vitek MIC results			Errors		
		*S*	ATU	*R*	*S*	*I*	*R*	VME	ME	MinE
*S. aureus* (*n* = 197)	Cefoxitin	92.4% (182/197)	≤ 1% (1/197)	7.1% (14/197)	92.9% (183/197)	/	7.1% (14/197)	/	/	/
*Enterococcus* spp. (*n* = 91)	Ampicillin	44.0% (40/91)	/	56.0% (51/91)	47.3% (43/91)	/	52.7% (48/91)	/	7.0% (3/43)	/
	Vancomycin	69.2% (63/91)	2.2% (2/91)	28.6% (26/91)	75.8% (69/91)	/	24.2% (22/91)	/	5.8% (4/69)	/
*E. coli* (*n* = 38)	Piperacillin/tazobactam	55.2% (21/38)	31.6% (12/38)	13.2% (5/38)	78.9% (30/38)	/	21.9% (8/38)	/	/	/
	Ciprofloxacin	50.0% (19/38)	23.7% (9/38)	26.3% (10/38)	65.8% (25/38)	10.5% (4/38)	23.7% (9/38)	/	/	6.9% (2/29)
	Meropenem	100% (38/38)	/	/	100% (38/38)	/	/	/	/	/
*K. pneumoniae* (*n* = 11)	Piperacillin/tazobactam	36.4% (4/11)	27.2% (3/11)	36.4% (4/11)	63.6% (7/11)	/	36.4% (4/11)	/	/	/
	Ciprofloxacin	72.7% (8/11)	/	27.3% (3/11)	81.8% (9/11)	/	18.2% (2/11)	/	11.1% (1/9)	/
	Meropenem	90.9% (10/11)	/	9.1% (1/11)	90.9% (10/11)	/	9.1% (1/11)	/	/	/
*P. aeruginosa* (*n* = 8)	Piperacillin/tazobactam	87.5% (7/8)	12.5% (1/8)	/	100% (8/8)	/	/	/	/	/
	Ciprofloxacin	62.5% (5/8)	25.0% (2/8)	12.5% (1/8)	87.5% (7/8)	12.5% (1/8)	/	/	14.3% (1/7)	/
	Meropenem	75.0% (6/8)	/	25.0% (2/8)	87.5% (7/8)	12.5% (1/8)	/	/	28.6% (2/7)	12.5% (1/8)

Overall categorical agreement was 96.5%. No VME was found in the RAST-MIC comparison. ME were found in 5.8% (4/69) and 7.0% (3/43) for *Enterococcus spp*. and vancomycin and ampicillin, 11.1% (1/9) and 14.3% (1/7) for *K. pneumoniae* and *P. aeruginosa* and ciprofloxacin, respectively. 28.6% (2/7) for *P. aeruginosa* and meropenem. 12.5% (1/8) and 6.5% (2/29) of MinE were found for *P. aeruginosa* and meropenem and *E.coli* and ciprofloxacin. Thirty out of 550 (5.5%) individual drug-species measurements were ATU (non for meropenem, 28.1% for piperacillin/tazobactam and 19.3% for ciprofloxacin).

**Fig. 2:**
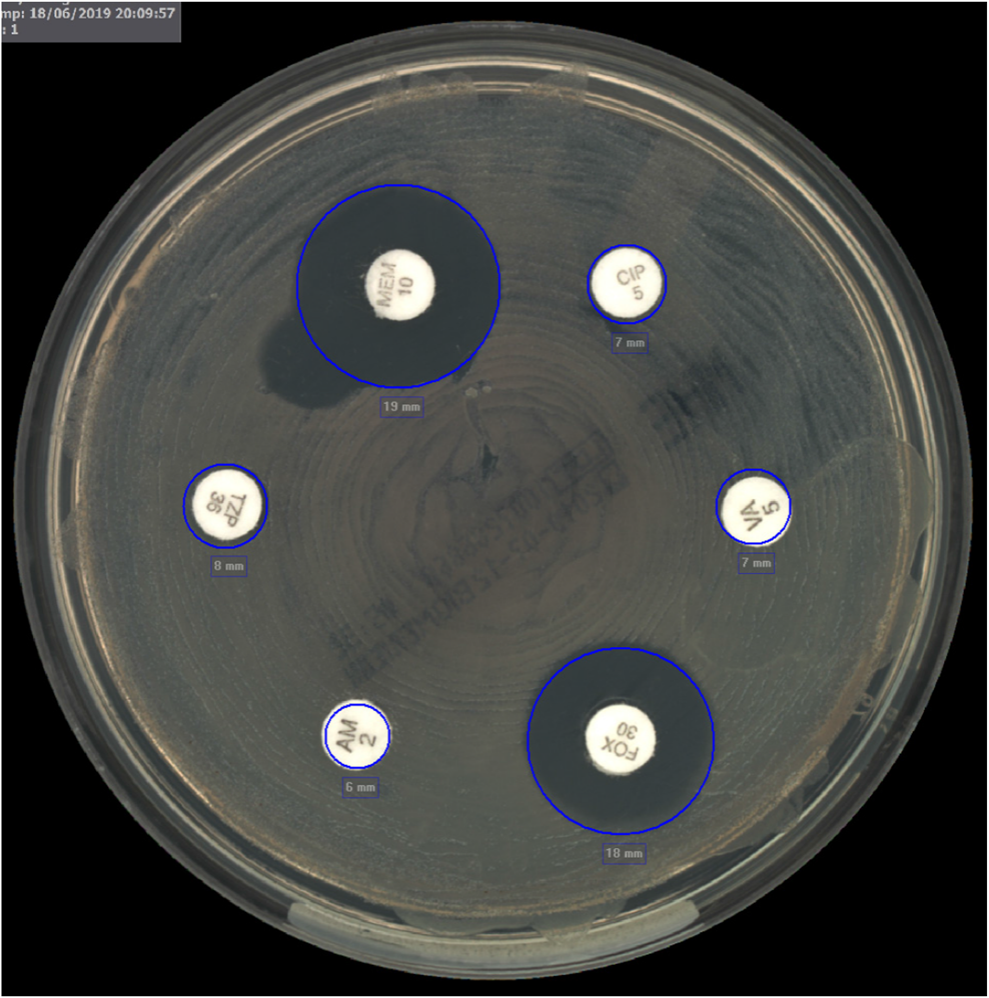
RAST image of bla_*OXA-48*_ carbapenemase-producing *K. pneumoniae* in drainage fluid inoculated in blood culture bottle at the Department for Infectious Diseases at the University Hospital Heidelberg, Germany. With a zone diameter of 19 mm, meropenem is susceptible according to the clinical breakpoints for RAST (version 1.1). MIC value of 1 mg/L obtained from Vitek2 confirmed the susceptible RAST result. bla_*OXA-48*_ carbapenemase was detected by PCR. The growth-free area outside the zone diameter of meropenem was due to manually correction of the antimicrobial plate after stamping the disks (CIP, ciprofloxacin; TZP, piperacillin/tazobactam, FOX, cefoxitin; VA, vancomycin; MEM, meropenem; AM, ampicillin)

Fourteen isolates of *methicillin-resistant S. aureus* (MRSA) were found by RAST and confirmed by MIC results. The same applied to 22 *vancomycin-resistant E. faecium* (VRE) isolates. An isolate of *K. pneumoniae* with *bla*_OXA-48_ was confirmed by PCR while RAST and MIC values reported susceptible response for meropenem (Fig. 2).

Data evaluation of drug-species combination showed no VME (Table 3). ME were found in ampicillin (7.0%), vancomycin (5.8%), ciprofloxacin (4.9%) and meropenem (4.3%). All exceeded the recommended 3% ME rate. MinE were detected for ciprofloxacin (4.3%) and meropenem (1.8%). Categorical agreement for all drug-species combination exceeded the recommended ≥ 90%.

**Table 3:** Errors and categorical agreement of 550 drug-species combination after comparing results of MIC values obtained from Vitek2 and zone diameters from rapid antimicrobial susceptibility testing (RAST) directly feasible on primarily sterile body fluids inoculated in blood culture bottles. For interpretation, EUCAST’s breakpoints for RAST (version 1.0, 1.1) for positive blood cultures were utilized on a total of 345 positive blood culture bottles inoculated with primarily sterile body fluids at the Department for Infectious Diseases at the University Hospital Heidelberg, Germany. Inhibition zones from RAST were compared to MIC results obtained from Vitek2. Very major errors (VME), major errors (ME) and minor errors (MinE) were calculated. RAST guidelines only contain an ‘area of technical uncertainty’ (ATU) where interpretation as susceptible, intermediate or resistant is not permitted. For determining VME and ME, isolates with ATU interpretation were excluded. MinE were only determined for susceptible and resistant RAST response to intermediate Vitek results (*S*, susceptible; *R*, resistant; *I*, susceptible, increased exposure; *ATU*, area of technical uncertainty)

	RAST interpretation			Vitek MIC results			Errors			Categorical agreement
	*S*	ATU	*R*	*S*	*I*	*R*	VME	ME	MinE	
Cefoxitin (*n* = 197)	92.4% (182/197)	≤ 1% (1/197)	7.1% (14/197)	92.9% (183/197)	/	7.1% (14/197)	/	/	/	99.5% (195/196)
Ampicillin (*n* = 91)	44.0% (40/91)	/	56.0% (51/91)	47.3% (43/91)	/	52.7% (48/91)	/	7.0% (3/43)	/	96.7% (88/91)
Vancomycin (*n* = 91)	69.2% (63/91)	2.2% (2/91)	38.6% (26/91)	75.8% (69/91)	/	24.2% (22/91)	/	5.8% (4/69)	/	94.4% (84/89)
Piperacillin/tazobactam (*n* = 57)	56.1% (32/57)	28.1% (16/57)	15.8% (9/57)	78.9% (45/57)	/	21.1% (12/57)	/	/	/	100% (41/41)
Ciprofloxacin (*n* = 57)	56.1% (32/57)	19.3% (11/57)	24.6% (14/57)	71.9% (41/57)	7.0% (4/57)	21.1% (12/57)	/	4.9% (2/41)	4.3% (2/46)	91.3% (42/46)
Meropenem (*n* = 57)	94.7% (54/57)	/	5.2% (3/57)	96.4% (55/57)	1.8% (1/57)	1.8% (1/57)	/	4.3% (2/55)	1.8% (1/57)	94.7% (54/57)

## Discussion

On a global scale, most sepsis deaths have an infectious cause which is why finding the source of infection is an important aim in the field of medical microbiology in order to treat properly. To do so, not only the identification of causative pathogens but also a faster and reliable AST has to be provided. Our current study focused on the rapid reporting of ID and AST by applying EUCAST’s RAST on primarily sterile body fluids sent in a blood culture bottle to see if RAST is applicable and can predict our final results.

As shown, we did not obtain any VME, only 11 ME and 3 MinE among 520 interpretable measurements. Though our species related errors exceeded the suggested rates by Cumitech, low denominators particularly regarding gram-negative rods like *P. aeruginosa* have to be considered. Similar problems with low denominators on blood-based blood culture bottles were recently discussed in a study on RAST by Soo et al. [12]. Furthermore, a larger number of isolates particularly more drug-resistant isolates would be useful to further evaluate the RAST method. Except the aforementioned problem with low denominators, the non-interpretation of ATU has to be considered for high species related errors as well. As already discussed by Jonasson et al. an unavoidable variation exists due to early reading, which is buffered by ATU and reduces VMEs and MEs [11]. Since challenging isolates for the establishment of RAST were used by EUCAST, our ATU fraction may be smaller due to the low level of multi-drug resistance [11]. A recent RAST study on Enterobacteriaceae by Martins et al. in Brazil showed an overall ATU of 21.6% for piperacillin/tazobactam and 5.3% for ciprofloxacin, respectively, which is lower compared to our current results [13]. Only meropenem (5.1%) displayed a clearly higher ATU fraction while our study did not have any ATUs in meropenem. If considered, that Brazil is expected to have a higher resistance level particularly in carbapenems, this result is comprehensible. Despite, the comparison is limited as only *E. coli* and *Klebsiella* spp. were considered in the study by Martins et al., while our study included *P. aeruginosa*. Furthermore, it remains unclear, if other species except *K. pneumoniae* were considered in Martins et al. study since the study mentions *Klebsiella* spp., though RAST is only accredited for *K. pneumoniae* [13, 14]. Also, EUCAST has evaluated a delayed RAST of up to 3 h for positive blood cultures kept at room temperature. Martins et al. considered RAST results with a delay of 4 ± 1 h [13, 14].

However, the 6 h reading is of limited benefit if a major part of zone diameters falls into ATU which particularly regards to settings of high ESBL prevalence [15]. In fact, our study revealed an ATU fraction of 28.1% for piperacillin/tazobactam and 19.3% for ciprofloxacin which is less compared to the study by Soo et al. [12]. Despite, ATU are not reported. Hence, clinician’s antimicrobial choice and a potential switch solely depend on the patient’s symptoms and laboratory results meaning a great loss of the RAST intention. Compared to our blood-based RAST study both antimicrobials have less errors and ATU leading to the presumption that blood may hamper appropriate reading or growth of gram-negative pathogens [8]. Results for Gram-positive cocci were similar for both studies. To our knowledge, no study on pathogen-blood interaction exists yet and consequently such a probable interaction remains interesting.

To reduce errors, EUCAST has recently updated the clinical breakpoints for *P. aeruginosa* and piperacillin/tazobactam and ciprofloxacin, respectively, by raising susceptible breakpoints to ≥ 50 mm. Zone diameters greater than ATU (13-15 mm) but smaller than susceptible are suggested to be interpreted as ‘susceptible, increased exposure’ [16]. However, with that EUCAST correction ME rates, which were more frequent not only in our blood and non-blood-based RAST studies, but also in studies conducted by Martins et al. and Soo et al., are not improved [12, 13]. Hence, an adaption of the resistant instead of susceptible zone diameters may rather address the problem.

Another error-prone fact was the measurement of zone diameters by numerous technicians. Though software was used, manual measurement with a difference of only 1 mm may lead to S/ATU/R. To minimize the observer variance, reading by a single experienced technician could be introduced which was done in a recent study with TLA [17]. However, this method is not feasible in our laboratory routine so that automatic inhibition zone reading could be a possibility. Though, automatic reading by OSIRIS system led to a slightly lower overall agreement and was additionally hampered by a poor growth particularly for *enterococci* compared to manual reading [18]. Indeed, light growth and unreadable zone diameters also occurred in a study conducted by CLSI, where 92.3% of *P. aeruginosa* were unreadable after 6 h indicating that breakpoints for these species may not be appropriate for an early read [19]. However, it is supposed that the use of a smart incubator system like TLA increases the readability of short-incubation disk diffusion method which is why we seldom had the problem of illegibility [19]. In the current study, only 4/20 (20.0%) isolates of *P. aeruginosa* could not be read (8 isolates had to be excluded from the study due to missing antimicrobial disks and technical issues). Furthermore, it has been assumed that short-incubation zone diameters of resistant isolates were either smaller or larger compared to 18 h reading leading to unpredictability [15].

Variation not only in measurement but also in the inoculum size may mislead interpretation, e.g. quantitating the number of organisms present in 1.0 ml of 10 randomly selected blood cultures resulted in an inoculum size ranging from 2 × 10^6^ to 6 × 10^7^, with a mean of 1.5x10^7^organisms/ml [20]. Even a recent study attributed discrepancies between direct testing and reference disk diffusion to the various bacterial concentrations and evaluated three commercial systems spanning nearly 3 logs [19]. Consequently, certain errors cannot be avoided. As Martins et al. have shown nicely, an increased inoculum size can only be compensated with an increased agar plate size resulting in 1:1 comparable zone diameters [13].

In our study we isolated one *bla*_*OXA-48*_ carrying isolate of *K. pneumoniae* which could neither be detected by Vitek2 nor by RAST. Similar results were found by Fröding et al. leading to the conclusion that carbapenemase cannot be detected sufficiently by 6 h reading [15]. In fact, our study highlights the well-known challenge for laboratories of detecting *bla*_*OXA-48*_ and again underlines the importance of additional molecular testing [12, 21].

Besides species with available clinical breakpoints for RAST we found a broad spectrum of other pathogens. Among them, *Staphylococcus epidermidis* (*n* = 211) was a frequent detected pathogen. Though pathogenicity of this bacterium is often unclear, we believe RAST would be beneficial for this certain pathogen or generally for *coagulase-negative staphylococci*. Considering this, we created a histogram comparing zone diameters from RAST to MIC results to show that breakpoints from *S. aureus* may not be applied on other *staphylococci* (Supplement 1).

A limitation of our study is our reference method. We used Vitek2 instead of broth microdilution because Vitek2 is the MIC determination method of our choice during the routine. Additionally, several papers already demonstrated that Vitek2 results are comparable to broth microdilution results [22–25]. Eventually, our aim was to demonstrate that RAST directly from positive blood culture bottles filled with primarily sterile body fluids are comparable to Vitek2 results so we can use RAST results for early reporting.

Another limitation was seen in poly-microbial findings (6.8%) where RAST could not be applied and Vitek2 results after isolation had to be awaited.

The integration of RAST with primarily sterile body fluids in antibiotic stewardship programs remains of certain interest and we intend to conduct such a clinical trial.

## Conclusion

Our study proved the applicability of RAST on various primarily sterile body fluids in blood culture bottles responding in a good categorical agreement between RAST-Vitek2, partially better than in blood-based RAST. This aim was supported by the optimal incubation in a TLA system leading to improved and punctual measurement of zone diameters.

Despite, an official evaluation with broth microdilution and recommendation by EUCAST is required. Our results support a buffer zone in form of ATU to avoid MEs or VMEs with further improvement of clinical breakpoints.

## Supplementary information

ESM 1 (DOCX 38 kb)
