# Supplementary material for: Applicability and performance of EUCAST’s rapid antimicrobial susceptibility testing (RAST) on primarily sterile body fluids in blood culture bottles in laboratory routine with total lab automation
Source: Eur J Clin Microbiol Infect Dis. 2021 Jan 12;40(6):1217–25. doi: 10.1007/s10096-020-04146-6 (PMC8139921; doi:10.1007/s10096-020-04146-6)
Supplement: Supplementary file 1 — (DOCX 38 kb) [file 10096_2020_4146_MOESM1_ESM.docx]

# Supplement 1

**R< 15mm**

**S≥ 18mm**

**ATU=17mm**

**Supplement 1 Histogram on zone diameters obtained from rapid antimicrobial susceptibility testing (RAST) compared to MIC distribution for *Staphylococcus epidermidis* and cefoxitin (n=119) in a study on RAST applied for primarily sterile body fluids inoculated in blood culture bottles at the Department for Infectious Diseases at the University Hospital Heidelberg, Germany.** Zone diameters were measured by technicians with a software function of Total Lab Automation (TLA). Zone diameters were then compared to MIC values and its interpretation obtained from Vitek2. The dashed lines display the clinical breakpoints for short incubation for *Staphylococcus aureus* and cefoxitin (according to zone diameter breakpoints for RAST Version 1.0 and 1.1 by EUCAST)*.*
(S=susceptible; R=resistant; ATU=area of technical uncertainty)
